# Effects of the PPAR-β agonist GW501516 in an *in vitro *model of brain inflammation and antibody-induced demyelination

**DOI:** 10.1186/1742-2094-6-15

**Published:** 2009-05-07

**Authors:** Antoinette Defaux, Marie-Gabrielle Zurich, Olivier Braissant, Paul Honegger, Florianne Monnet-Tschudi

**Affiliations:** 1 Department of Physiology, University of Lausanne, CH-1005 Lausanne, Switzerland; 2 Clinical Chemistry Laboratory, Centre Hospitalier Universitaire Vaudois and University of Lausanne, CH-1011 Lausanne, Switzerland

## Abstract

**Background:**

Brain inflammation plays a central role in numerous brain pathologies, including multiple sclerosis (MS). Microglial cells and astrocytes are the effector cells of neuroinflammation. They can be activated also by agents such as interferon-γ (IFN-γ) and lipopolysaccharide (LPS). Peroxisome proliferator-associated receptor (PPAR) pathways are involved in the control of the inflammatory processes, and PPAR-β seems to play an important role in the regulation of central inflammation. In addition, PPAR-β agonists were shown to have trophic effects on oligodendrocytes *in vitro*, and to confer partial protection in experimental autoimmune encephalomyelitis (EAE), an animal model of MS. In the present work, a three-dimensional brain cell culture system was used as *in vitro *model to study antibody-induced demyelination and inflammatory responses. GW 501516, a specific PPAR-β agonist, was examined for its capacity to protect from antibody-mediated demyelination and to prevent inflammatory responses induced by IFN-γ and LPS.

**Methods:**

Aggregating brain cells cultures were prepared from embryonal rat brain, and used to study the inflammatory responses triggered by IFN-γ and LPS and by antibody-mediated demyelination induced by antibodies directed against myelin-oligodendrocyte glycoprotein (MOG). The effects of GW 501516 on cellular responses were characterized by the quantification of the mRNA expression of tumor necrosis factor-α (TNF-α), interleukin-6 (IL-6), inducible NO synthase (i-NOS), PPAR-β, PPAR-γ, glial fibrillary acidic protein (GFAP), myelin basic protein (MBP), and high molecular weight neurofilament protein (NF-H). GFAP expression was also examined by immunocytochemistry, and microglial cells were visualized by isolectin B4 (IB4) and ED1 labeling.

**Results:**

GW 501516 decreased the IFN-γ-induced up-regulation of TNF-α and iNOS in accord with the proposed anti-inflammatory effects of this PPAR-β agonist. However, it increased IL-6 m-RNA expression. In demyelinating cultures, reactivity of both microglial cells and astrocytes was observed, while the expression of the inflammatory cytokines and iNOS remained unaffected. Furthermore, GW 501516 did not protect against the demyelination-induced changes in gene expression.

**Conclusion:**

Although GW 501516 showed anti-inflammatory activity, it did not protect against antibody-mediated demyelination. This suggests that the protective effects of PPAR-β agonists observed *in vivo *can be attributed to their anti-inflammatory properties rather than to a direct protective or trophic effect on oligodendrocytes.

## Background

Neuroinflammation is a common phenomenon in numerous brain pathologies [1]. In multiple sclerosis (MS), active demyelinating lesions are surrounded by inflammatory foci [2,3]. Inflammation plays a central role in MS pathology, contributing to both the onset and the progression of this autoimmune disease. Brain inflammatory reactions involve microglial cells and astrocytes, which in the activated state undergo profound changes in cell morphology and physiology, accompanied by the release of numerous inflammatory mediators and other bioactive factors [4-6]. Among the pro-inflammatory mediators, tumor necrosis factor-α (TNF-α) and interleukin-6 (IL-6) seem to play a predominant role because of their involvement at multiple levels of neuroimmune regulation (for review [5,7]). An inflammatory response can be induced experimentally by various agents activating microglial cells and astrocytes. The cytokine interferon-γ (IFN-γ), secreted by activated lymphocytes and detected in the brain during the symptomatic phase of MS [8], can directly activate cells of the macrophage lineage [9-11]. IFN-γ is also able to target oligodendrocytes [12] and can lead to demyelination after a repeated application [13]. The endotoxin lipopolysaccharide (LPS) from bacterial origin has been shown to activate microglia and to induce the expression of pro-inflammatory mediators [14,15]. LPS also induced the death of oligodendrocytes and neurons [16,17].

Among the peroxisome proliferator activated receptors (PPARs), a family of nuclear transcription factors, PPAR-β, also known as PPAR-δ, FAAR and NUC-1, is the predominant and most widely expressed subtype in the brain. It is highly expressed in the developing neural tube [18] as well as in oligodendrocytes and neurons of the adult brain [19,20]. Nevertheless, still little is known about its physiological ligands [21,22] and target genes [23]. PPARs, and particularly PPAR-β, are supposed to modulate brain cell maturation, which may involve also inflammatory mediators released by brain cells [24]. PPAR-β activity promoted oligodendrocyte development and myelin formation [25-27], and PPAR-β deficient mice showed altered myelination [28]. Besides their involvement in metabolism, and in particular in lipid metabolism (for review [29]), PPARs, when activated, are able to trans-repress the activation of the NF-κB pathway [30,31] which decreases inflammatory gene expression [31-38]. The anti-inflammatory effects of PPAR-γ and PPAR-α agonists are well described. Specific agonists of PPAR-α [39] and PPAR-γ [36,37] were found to inhibit the release of pro-inflammatory cytokines by microglial cells and astrocytes, and to be effective in the treatment of experimental autoimmune encephalomyelitis (EAE), an animal model of MS [40,41]. Moreover a PPAR-γ agonist was shown to partially protect aggregating brain cell cultures from antibody-induced demyelination [42]. Concerning the anti-inflammatory potential of PPAR-β, little is known. Nevertheless, a protective effect in EAE was also reported for a PPAR-β-specific agonist [43]. Furthermore, PPAR-β is supposed to play an important role in the control of central inflammation, as indicated by an increased infarct size, and an increase in the level of interferon-γ in PPAR-β KO mice compared to wild-type mice [44] in a model of focal cerebral ischemia. Therefore, it is thought that PPAR agonists could be used therapeutically as potent anti-inflammatory agents.

In the present work, aggregating brain cell cultures were used as *in vitro *model to study the effects of GW 501516, a specific PPAR-β agonist, on brain inflammation and on antibody-induced demyelination. These 3-dimensional cell cultures were prepared from mechanically dissociated embryonal brain cells and grown in a chemically defined medium [45]. Under constant gyratory agitation, free-floating spheroids of 200–300 μm diameter form spontaneously, allowing a high degree of cell-cell interactions and extensive neuronal and glial maturation. Within the aggregates, the different brain cell types (i.e., neurons, astrocytes, oligodendrocytes, and microglia) are organized histotypically, while lymphocytes and fibroblasts are absent, providing a unique model to study the anti-inflammatory potential of PPAR-β on brain inflammation. For experimentation, aggregates were taken at culture day 26 (DIV 26), when the myelination of axons was nearly maximal. The inflammatory response was triggered by the treatment with IFN-γ and LPS. Antibody-mediated demyelination was induced as described previously [13,46,47]. The present results show that GW 501516 was efficacious as anti-inflammatory agent, but did not protect oligodendrocytes against antibody-induced demyelination in this *in vitro *model.

## Methods

### Aggregating brain cell cultures

Serum-free aggregating brain cell cultures were prepared from the telencephalon of 16-day embryonal rats (Hsd:SD, Harlan, NL-5960 AD Horst) as described previously in detail [45,48]. The embryonal brain tissue was mechanically dissociated using nylon sieves of 100-μm and 200-μm pores, and the dissociated cells were incubated under gyratory agitation in serum-free medium. The resulting aggregate cultures were maintained in serum-free medium under constant gyratory agitation (80 rpm) at 37°C in an atmosphere of 10% CO_2 _and 90% humidified air. Media were replenished by the replacement of 5 ml of culture supernatant (of a total of 8 ml per flask) with fresh medium every 3^rd ^day until day *in vitro *(DIV) 14, and every 2^nd ^day thereafter.

### Antibody-mediated demyelination

Antibody-mediated demyelination was performed as described previously [13,46,47]. At DIV 26, culture replicates were prepared by randomizing and aliquoting the aggregates of the original cultures. The aggregates from several flasks were pooled, and aliquots of the aggregate suspension redistributed into flasks containing pre-equilibrated medium (to give a total volume of 4 ml). Two sets of control cultures were used, one that remained untreated, and another that received guinea pig serum (25 μl/ml) as a source of complement. Demyelination was induced by the addition of guinea pig serum (25 μl/ml) and rat anti-MOG antibodies (62.5 μg/ml). This antibody was derived from clone 8–18C5 [47]. The immunoglobulin G (IgG) fraction was purified by affinity chromatography using the Bio-Rad Econo-Pac protein A kit (Bio-Rad, Richmond, CA, USA).

### Chemicals

All chemicals used were of the highest available purity. GW 501516 (Alexis Biochemicals) was dissolved in dimethylsulfoxid (DMSO), and 10^3^-fold concentrated stock solutions were prepared and stored at 4°C, protected from light. The final concentration of DMSO in treated cultures and controls was 0.1% (v/v). Interferon-γ (Peprotec) (50 U/ml) was dissolved in phosphate buffered saline (PBS) supplemented with 0.1% BSA (pH 8). Lipopolysaccharide (LPS, 5 μg/ml) (Sigma) was dissolved in sterile NaCl (0.9% w/v).

### Biochemical assays

For biochemical analyses, brain cell aggregates were washed twice with 5 ml of ice-cold PBS and homogenized in 0.4 ml of potassium phosphate buffer (2 mM, pH 6.8) containing 1 mM EDTA, using glass-teflon homogenizers (Bellco, Vineland, NJ, USA). The different homogenates were briefly sonicated and stored in aliquots for the different assays at -80°C. The protein concentration was determined by the Folin phenol method [49] using bovine serum albumin as standard. The intracellular lactate dehydrogenase (LDH; EC 1.1.1.27) activity was measured photometrically [50] to assess cytotoxicity.

### Quantitative RT-PCR

Aggregating cell cultures were washed twice with 5 ml of ice-cold PBS and stored at -80°C in RNA later (Qiagen AG, Basel, Switzerland). The RNeasy kit from Qiagen was used to extract total RNA. The reverse transcription (RT) reaction was performed using the High capacity cDNA Reverse Transcription Kit and protocols from Applied Biosystem (ABI, Foster City, CA, USA). Briefly, the RT was run with 2 μg of total RNA in a reaction volume of 20 μl. Aliquots of this reaction mixture were used for the subsequent PCR reactions. The PCR mixture (10 μl) was composed of primers (150–400 mmol/l), 1× SYBR Green PCR master mix (ABI) and H_2_O. For measuring the expression of iNOS, MBP, MOG, NF-H, PPAR-β and PPAR-γ, 3.2 ng of cDNA was disposed per well. For the expression of IL-6 and TNF-α, 16 ng of cDNA was disposed in each well. Each set of primer sequences was designed to meet the quality criteria previously described in detail [51]. Results are calculated using the ΔCt method [52]. Results are expressed as fold change relative to untreated control cultures, each value coming from 6–7 replicate cultures obtained in 2 independent experiments performed with cultures from different batches. The following sequences were used: GFAP, forward: CCT TGA CCT GCG ACC TTG AG, reverse: GCG CAT TTG CCT CTC ACA CAG A; IL-6, forward: ATA TGT TCT CAG GGA GAT CTT GGA A, reverse: TGC ATC ATC GCT GTT CAT ACA A; iNOS, forward: TCC TCA GGC GGT CTT GTT A, reverse: CTG CAC CAA CTC TGC TGT TCT C; MBP, forward: GCA CGC TTT CCA AAA TCT TTA AG, reverse: AGG GAG GC TCT CAG CGT CTT; MOG, forward: TGT AGG CCT TGT ATT CCT CTT CCT, reverse: TCC GAT GGA GAT TCT CGA CTT C; NF-H, forward: CAG GAC CTG CTC AAC GTC AA, reverse: CTT CGC CTT CCA GGA GTT TTC T; PPAR-β, forward: AGA ACC GCA ACA AGT GTC AGT ACT, reverse: CTC CGG CAT CCT TCC AAA G; PPAR-γ, forward: GAC CCA ATG GTT GCT GAT TAC A, reverse: GGG ACG CAG GCT CTA CTT TG; TNF-α, forward: ACC CTC ACA CTC AGA TCA TCT TC, reverse: TGG TGG TTT GCT ACG T

### Immunocytochemistry and *in situ *hybridization

Aggregating brain cell cultures used for immunocytochemistry and *in situ *hybridization were washed twice with prewarmed PBS, embedded in cryomatrix (Jung, Nussloch, Germany), frozen in isopentane cooled with liquid nitrogen, and stored at -80°C [48].

For immunocytochemistry, cryosections (10 μm) were fixed for 10 minutes in 4% paraformaldehyde in PBS at room temperature, washed in PBS, and kept overnight at 4°C. Sections were incubated first in horse serum (1:25 in PBS with 0.1% Triton-X100, Vector) for blockade of non-specific binding, then exposed overnight at 4°C to a monoclonal antibody directed against GFAP (1:800; Sigma). For staining, biotinylated horse anti-mouse IgG (1:200; Vector) and avidine coupled to FITC (avidine DCS, Vector) were used. Sections were mounted in Vectashield with DAPI (Vector Laboratories) and analyzed on a Zeiss LSM 510 Meta confocal microscope.

For IB4 labeling, microglia were visualized in sections of PBS-washed and Carnoy-fixed aggregates by the specific binding of horseradish peroxidase-conjugated lectin (GSI-B4) of *Griffonia simplicifolia *according to Streit and Kreutzberg [53] and Ashwell [54]. Briefly, aggregates were fixed in Carnoy and embedded in paraplast. The 5 μm sections were incubated for 30 min in absolute methanol containing 0.3% H_2_O_2 _to block endogenous peroxidase activity, and then exposed overnight at 4°C to the horseradish peroxidase-conjugated lectin (*Griffonia simplicifolia *GSI-B4 isolectin, conjugated with type VI HRP, Sigma) dissolved at a final concentration of 1.25 mg per 100 ml in 0.1 M of Tris-buffered saline (pH 7.4) containing 1% Triton X-100. As a control, the specific lectin binding sites were saturated by preincubation (2 h at room temperature) with 0.1 M of melibiose (6-O-α-D-galactopyranosyl-D-glucose, Sigma). Quantification of microglial staining was performed using Image J.

For *in situ *hybridization, a cDNA comprising nucleotides 1–238 of the sequence of rat PPAR-β (Genebank AJ306400; [55]) was subcloned into the BamHI and SmaI sites of the pBluescript KS^- ^vector (Stratagene, Heidelberg, Germany), yielding pBS-PPAR-β. Digoxigenin labelled PPAR-β riboprobes were transcribed *in vitro *as described [56]. The antisense probe was transcribed from pBS-PPAR-β linearized with XbaI, while the sense probe was synthesized from pBS-PPAR-β linearized with HindIII. Of the frozen aggregating brain cell cultures, cryosections (12 μm) were prepared, and analyzed by *in situ *hybridization as described [56]. Briefly, hybridization with antisense and sense riboprobes for rat PPAR-β was carried out at 58°C in 5 × SSC and 50% formamide for 40 hours. Then, washes (30 minutes in 2 × SSC at room temperature, 1 hour in 2 × SSC at 65°C, 1 hour in 0.1 × SSC at 65°C), and alkaline-phosphatase staining (15 hours at room temperature) were performed. The specificity of hybridization was ascertained by the use of a sense probe having the same length, GC content, and specificity as the antisense probe. Sections were further processed for immunohistochemistry as described [56]. Neurons, astrocytes, oligodendrocytes, and microglia were labelled using anti-MAP2 (mouse monoclonal, MAB378, Chemicon), GFAP (mouse monoclonal, MAB3402, Chemicon), MBP (goat polyclonal, sc-13914, Santa Cruz), ED1 (mouse monoclonal, Santa Cruz Biotechnology) antibodies and GSI-B4, respectively. Briefly, after rehydration, the ISH stained sections were fixed 1 h in 4% paraformaldehyde-PBS at room temperature and washed 3 × 5 min in PBS. Immunohistochemistry was then performed with primary antibodies diluted 1:100 for MAP2 and GFAP, and 1:50 for ED1, and subsequently with the mouse Histostain-Plus kit (Zymed Laboratories); and for MBP by anti-goat IgG biotinylated secondary antibody followed by streptavidin-peroxidase conjugation. Peroxidase staining was performed for 10 min using aminoethyl carbazole (AEC) and H_2_O_2_. The double-stained sections (blue signal for ISH and red signal for immunohistochemistry) were mounted in glycerol.

### Statistics

For mRNA expression, results are expressed as fold change compared to untreated control cultures. Statistical evaluations were made by the Kruskal-Wallis test followed by the Mann-Whitney test.

## Results

### Effects of GW 501516 in IFN-γ- and LPS-induced inflammatory responses

Aggregating brain cell cultures were treated at DIV 26 with IFN-γ (50 U/ml) and LPS (5 μg/ml), given either separately or combined. GW 501516 (5 μM) was added twice, first 18 hours before, and then simultaneously with the inflammatory agent(s). The concentration of GW 501516 chosen (5 μM) was based on a previous concentration-response evaluation between 0.1 μM to 10 μM at different developmental periods, showing in mature cultures the absence of cytotoxicity up to 5 μM, as assessed by the measurement of LDH activity (data not shown).

**Figure 1:**
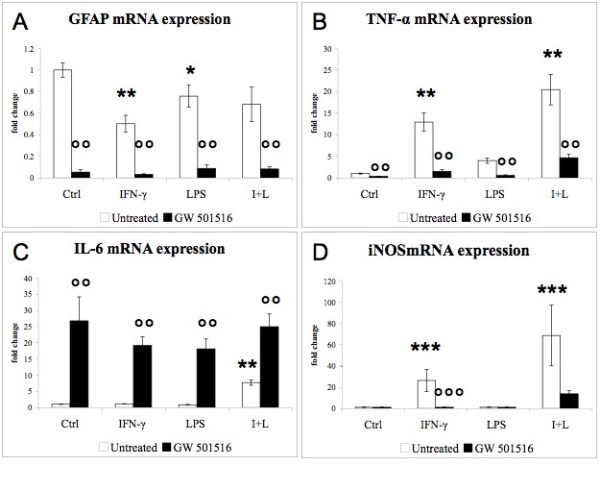
**Effects of IFN-γ, LPS and GW 501516 on GFAP, TNF-α, IL-6, and iNOS mRNA expression**. GW 501516 down-regulated GFAP mRNA expression in control cultures and in cultures treated with the inflammatory agents (A). GW 501516 decreased the up-regulation of TNF-α induced by the inflammatory agents. GW 501516 up-regulated IL-6 expression in control cultures and in cultures treated with the inflammatory agents (C), and it decreased the IFN-γ-induced up-regluation of iNOS expression (D). Cultures received GW 501516 (5 μM) 18 hours before the addition of the inflammatory agents, and again together with INF-γ (50 U/ml) and LPS (5 μg/ml). Cultures were harvested 48 hours after the inflammatory treatment. Values are expressed as fold change relative to the untreated control cultures (= 1), each value being the mean of 7 replicate cultures. Results were statistically evaluated for significance by the Kruskal-Wallis test followed by the Mann-Whitney test. (* P < 0.05, **P < 0.01,***P < 0.001 compared with untreated control cultures; °°P < 0.01, °°°P < 0.001 compared with cultures not treated with GW 501516).

The inflammatory responses were examined 48 hours after the addition of the inflammatory agents. GFAP mRNA expression (Fig. 1A) was significantly decreased by IFN-γ and LPS, while GFAP immunostaining remained unchanged after the treatment with either IFN-γ or LPS (data not shown). GW 501516 decreased GFAP mRNA expression in control cultures, and this decrease was also observed in the presence of the inflammatory agents (Fig 1A, black bars). TNF-α mRNA expression was greatly increased in response to either IFN-γ or LPS (Fig. 1B, white bars), and further increased in the presence of both agents. GW 501516 strongly reduced the IFN-γ- and LPS-induced up-regulation of TNF-α (Fig 1B, black bars). IL-6 mRNA expression was up-regulated by the combined treatment with IFN-γ and LPS but not by the separate treatments with these agents (Fig. 1C, white bars). GW 501516 increased IL-6 expression in control cultures and in cultures treated with IFN-γ and LPS (Fig. 1C, black bars). The expression of iNOS mRNA (Fig. 1D) was strongly up-regulated by IFN-γ and by the combined treatment with IFN-γ and LPS, but not by LPS alone. GW 501516 greatly decreased the IFN-induced up-regulation of iNOS. Besides the up-regulation of the expression of cytokines and iNOS, IFN-γ and LPS increased the number and the clustering of microglia (data not shown), indicating microglial activation.

**Figure 2:**
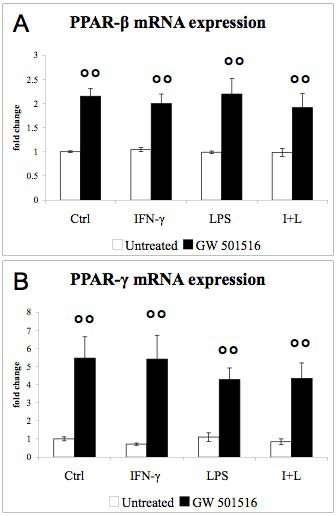
**Effects of IFN-γ, LPS, and GW 501516 on PPAR-β and PPAR-γ mRNA expression**. GW 501516 (black bars) up-regulated the expression of PPAR-β (A) and PPAR-γ (B) in control cultures and in cultures treated with the inflammatory agents. Cultures received GW 501516 (5 μM) 18 hours before the addition of the inflammatory agents, and again together with INF-γ (50 U/ml) and LPS (5 μg/ml). Cultures were harvested 48 hours after the inflammatory treatment. Values are expressed as fold change relative to the untreated control cultures (= 1), each value being the mean of 7 replicate cultures. Results were statistically evaluated for significance by the Kruskal-Wallis test followed by the Mann-Whitney test. (°°°P < 0.01 compared with cultures not treated with GW 501516).

**Figure 3:**
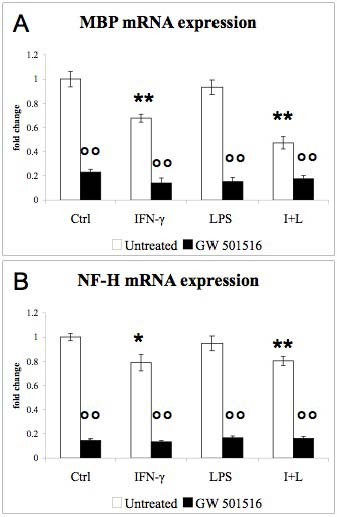
**Effects of IFN-γ, LPS, and GW 501516 on MBP and NF-H mRNA expression**. GW 501516 (black bars) decreased MBP (A) and NF-H (B) mRNA expression in control cultures and in cultures treated with the inflammatory agents. Cultures received GW 501516 (5 μM) 18 hours before the addition of the inflammatory agents, and again together with INF-γ (50 U/ml) and LPS (5 μg/ml). Cultures were harvested 48 hours after the inflammatory treatment. Values are expressed as fold change relative to the untreated control cultures (= 1), each value being the mean of 7 replicate cultures. Results were statistically evaluated for significance by the Kruskal-Wallis test followed by the Mann-Whitney test. (* P < 0.05, **P < 0.01 compared with untreated control cultures; °°P < 0.01 compared with cultures not treated with GW 501516).

PPAR-β and PPAR-γ mRNA levels (Fig 2A, B) remained unaffected by IFN-γ and LPS, but were up-regulated in response to GW 501516. Furthermore, IFN-γ decreased MBP (Fig 3A) as well as MOG (data not shown) mRNA expression. LPS alone did not affect MBP expression, while it increased the IFN-γ-induced drop in MBP mRNA (Fig 3A). NF-H mRNA expression was slightly down-regulated by IFN-γ but not affected by LPS (Fig 3B, black bars). GW 501516 strongly decreased MBP expression in control cultures as well as in cultures treated with the inflammatory agents (Fig 3A, black bars). GW 501516 also strongly decreased NF-H expression in both the presence and the absence of the inflammatory agents (Fig 3B, black bars).

### Effects of GW 501516 in antibody-induced demyelination

**Figure 4:**
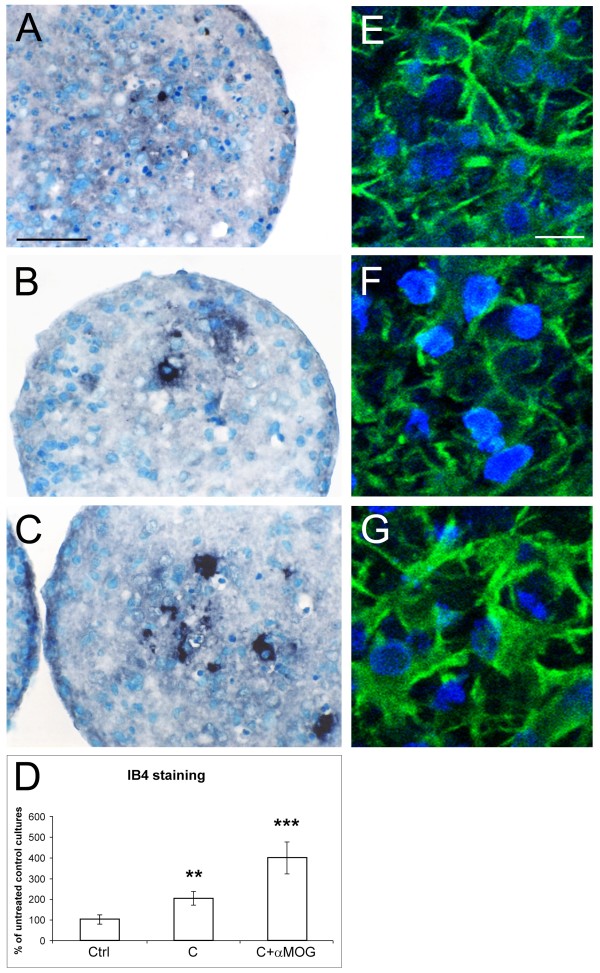
**Reactivity of microglial cells and astrocytes after antibody-mediated demyelination**. IB4-labeled microglial cells (A–C), 48 hours after the demyelinating insult, were more numerous in cultures subjected to the demyelinating treatment (C compared to A). Some of them contained vacuoles and were increased in size, suggesting a macrophagic state. Complement alone caused a slight microglial activation (B compared to A). Quantification of IB4-labeled microglial cells (D) expressing the labeled area as percent of untreated control cultures. Twenty aggregate sections per treatment were measured. Results were statistically evaluated for significance by the Kruskal-Wallis test followed by the Mann-Whitney test. (**P < 0.01, ***P < 0.001 compared with untreated control cultures). Astrocytes immunostained for GFAP (E–G) showed that demyelination caused enlarged astrocytic processes and increased immunostaining (G compared to E). Complement alone did not affect neither astrocytic morphology nor GFAP staining (F compared to E). A and E, untreated controls; B and F, complement treated (guinea pig serum, 25 μl/ml); C and G, treated with antibody (anti-MOG, 62.5 μg/ml) and complement. A–C: bar = 50 μm; E–G: bar = 10 μm.

**Figure 5:**
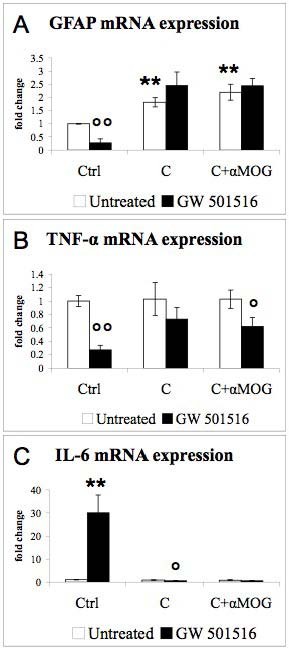
**Effects of antibody-mediated demyelination and GW 501516 on GFAP, TNF-α, and IL-6 mRNA expression**. The antibody-mediated demyelination induced a significant increase of GFAP mRNA (A), but did not affect TNF-α (B) nor IL-6 (C) mRNA expression. Cultures received GW 501516 (5 μM) 18 hours before and again together with the demyelinating agents. Cultures were harvested 48 hours after the demyelinating treatment. Values are expressed as fold change relative to the untreated control cultures (= 1), each value being the mean of 6 replicate cultures. Results were statistically evaluated for significance by using the Kruskal-Wallis test followed by the Mann-Whitney test (**P < 0.01 compared with untreated control cultures; °P < 0.05, °°P < 0.01 compared with cultures not treated with GW 501516).

The effects of GW 501516 were further investigated in an *in vitro *model of antibody-mediated demyelination. Aggregating brain cell cultures were treated at DIV 26 with anti-MOG antibodies (62.5 μg/ml) and complement (guinea pig serum, 25 μl/ml) to induce demyelination. Cultures were treated twice with 5 μM of GW 501516, first 18 hours before the induction of demyelination and then simultaneously with the demyelinating agents. The effects of the demyelinating treatment and of GW 501516 on several inflammatory markers were examined 48 hours after the induction of antibody-mediated demyelination, when MBP and MOG expression were decreased at both the mRNA and protein levels indicating myelin loss [42,47,57]. The reactivity of microglial cells and astrocytes in response to the antibody-mediated demyelination was first examined by morphological and immunocytochemical analyses. As shown in Fig. 4, 48 h after the demyelinating insult, the number of IB4-labeled microglial cells was significantly increased compared to the untreated controls (Fig. 4C*vs*. 4A, and Fig. 4D). Some of the microglial cells were increased in size and contained vacuoles, indicating a macrophage-like state. In cultures treated with complement alone, few microglial cells exhibited this reactive phenotype (Fig. 4B). Demyelinating cultures also showed enlarged astrocytic processes and increased intensity of GFAP immunostaining (Fig. 4F* vs*. 4D), suggesting a strong astrocytic reaction. In accord with this observation, GFAP mRNA levels were significantly increased (Fig. 5A, white bars). In cultures treated with complement alone, GFAP immunostaining (Fig 4E*vs*. 4D) appeared unchanged, while the GFAP mRNA levels were increased (Fig. 5A, white bars). These findings suggest that compared to the strong glial reactivity in response to the antibody-mediated demyelination, complement (i.e., guinea pig serum) alone caused a relatively weak glial response, in relation with its slight demyelinating effect as observed previously [13,58]. The presence of GW 501516 strongly decreased GFAP mRNA expression in control cultures, but did not modify the GFAP up-regulation in demyelinating cultures (Fig. 5A). The measurements of cytokine mRNA levels showed that TNF-α expression was not significantly modified by the demyelinating agents (Fig. 5B, white bars), while the treatment with GW501516 decreased significantly TNF-α expression in control cultures and in demyelinating cultures (Fig 5B, black bars). IL-6 mRNA expression (Fig 5C) was low in untreated cultures and in cultures treated with the demyelinating agents, while it was strongly increased in GW 501516-treated control cultures.

**Figure 6:**
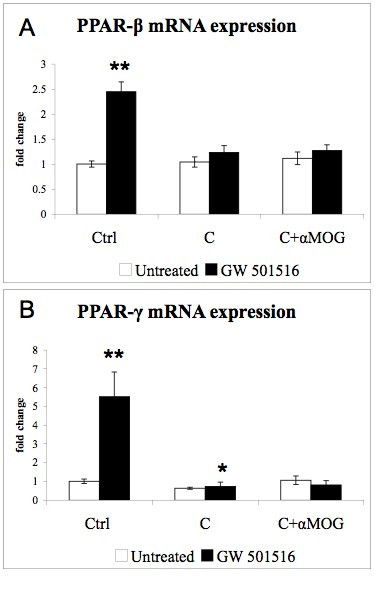
**Effects of antibody-mediated demyelination and GW 501516 on PPAR-β and PPAR-γ mRNA expression**. GW 501516 (black bars) up-regulated PPAR-β (A) and PPAR-γ (B) expression in control cultures but not in demyelinating cultures. Cultures were treated with GW 501516 (5 μM) 18 hours before and again together with the demyelinating agents. Cultures were harvested 48 hours after the demyelinating treatment. Values are expressed as fold change relative to the untreated control cultures (= 1), each value being the mean of 6 replicate cultures. Results were statistically evaluated for significance by using the Kruskal-Wallis test followed by the Mann-Whitney test (* P < 0.05, **P < 0.01, compared with untreated control cultures).

**Figure 7:**
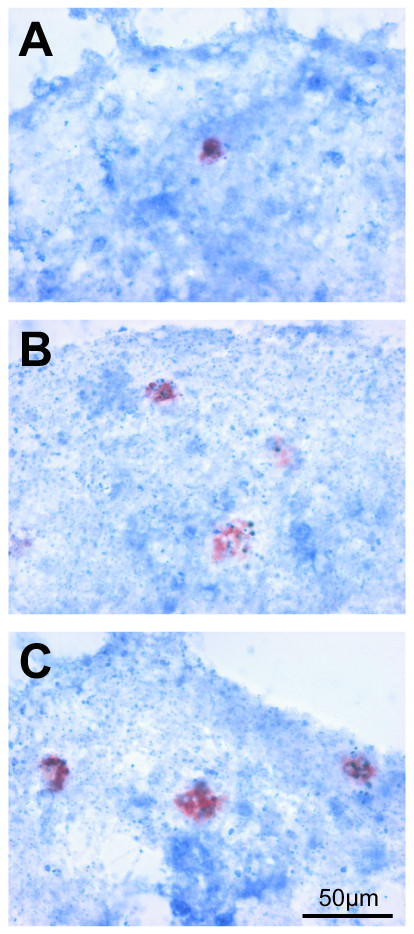
**Expression of PPAR-β mRNA in microglial cells after antibody-mediated demyelination**. The antibody-mediated demyelination did not modify the cellular expression of PPAR-β analyzed by in situ hybridization. Macrophagic microglial cells labeled by ED1 were more numerous in cultures subjected to the demyelinating treatment (C compared to A and B, respectively). A, untreated control; B, complement treated (guinea pig serum, 25 μl/ml); C, treated with antibody (anti-MOG, 62.5 μg/ml) and complement. Bar = 50 μm.

**Figure 8:**
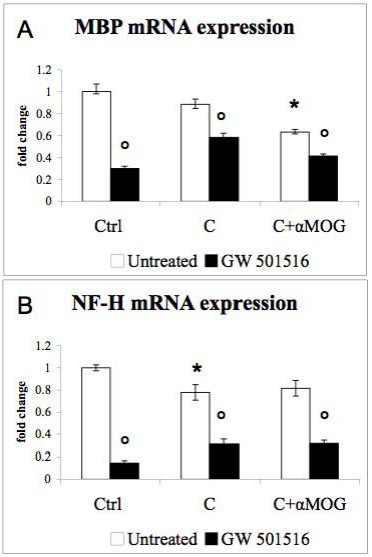
**Effects of antibody-mediated demyelination and GW 501516 on MBP and NF-H mRNA expression**. GW 501516 (black bars) decreased MBP (A), and NF-H (B) mRNA expression in control cultures and in demyelinating cultures. Cultures received GW 501516 (5 μM) 18 hours before and again together with the inflammatory agents. Cultures were harvested 48 hours after the demyelinating treatment. Values are expressed as fold change relative to the untreated control cultures (= 1), each value being the mean of 6 replicate cultures. Results were statistically evaluated for significance by the Kruskal-Wallis test followed by the Mann-Whitney test. (* P < 0.05 compared with untreated control cultures; °P < 0.05 compared with cultures not treated with GW 501516).

This increase did not occur in cultures which received complement alone or antibody plus complement. The levels of iNOS mRNA were not affected, neither by the demyelinating treatment nor by the treatment with GW 501516 (data not shown). Furthermore, the demyelinating treatment did not modify PPAR-β (Fig 6A) nor PPAR-γ (Fig 6B) mRNA expression. GW 501516 up-regulated the expression of PPAR-β (Fig 6A) and PPAR-γ (Fig 6B) in control cultures, but not in demyelinating cultures. The analysis by *in situ *hybridization indicated that PPAR-β was expressed by neurons as well as by glial cells (data not shown). Microglia immunolabeled by ED1 (Fig 7) were macrophagic and more numerous in cultures subjected to antibody-mediated demyelination, in accord with the results obtained by IB4 labeling (Fig 4). Furthermore, the demyelinating treatment did not modify the cellular expression of PPAR-β (Fig. 7, C compared to A and B, respectively). As expected, the demyelinating treatment decreased MBP mRNA expression (Fig. 8A). GW 501516 strongly down-regulated the mRNA expression of MBP in control cultures (Fig. 8A) as observed previously (Fig. 3A), and exacerbated the decrease of MBP mRNA in denyelinating cultures. NF-H expression (Fig 8B) was not affected by the demyelinating treatment, but by GW 501516, which decreased NF-H mRNA levels in controls and in demyelinating cultures. Nevertheless, the treatment with GW 501516 did not affect the LDH activity in these cultures (data not shown) indicating the absence of cytotoxicity.

## Discussion

The responsiveness of aggregating brain cell cultures to inflammatory stimuli and the anti-inflammatory effects of the specific PPAR-β agonist GW 501516 were investigated first by using two conventional inflammatory agents, IFN-γ and LPS. In good agreement with its known inflammatory activity, IFN-γ strongly up-regulated TNF-α and iNOS mRNA expression and caused microglial reactivity. It also decreased the expression of GFAP, MBP and NF-H at the mRNA level, without affecting cellular viability. The down-regulation of MBP mRNA expression by IFN-γ is in good agreement with previous observations [59]. In comparison to IFN-γ, LPS caused only a relatively weak inflammatory response, indicated by a moderate up-regulation of TNF-α, whereas the combined treatment with IFN-γ and LPS strongly up-regulated IL-6, TNF-α, and iNOS expression. Under these inflammatory conditions, GW 501516 clearly exhibited anti-inflammatory properties, since it strongly attenuated the up-regulation of TNF-α and iNOS. On the other hand, it greatly up-regulated the mRNA expression of IL-6. Since IL-6 is generally viewed as a pro-inflammatory cytokine, this finding seems to contradict the anti-inflammatory action of GW 501516. However, IL-6 is known to be a pleiotropic cytokine. It was shown to contribute to glial development and neuroprotection in the brain [60-64], whereas cerebral overexpression of IL-6 in astrocytes, and systemic administration of IL-6 together with its soluble receptor sIL-6Rα lead to neurodegeneration, gliosis, and microglial activation (for review [7]). Up-regulation of IL-6 was observed in neurons *in vivo *after excitotoxic damage [65]. The present finding that GW 501516 up-regulated IL-6 concomitantly with the down-regulation of NF-H may therefore indicate a detrimental effect of this PPAR-β agonist on neurons.

In macrophages, Welch and collaborators [38] have shown that the PPAR-β agonist GW 0742 decreased the LPS-induced up-regulation of iNOS and COX2. In addition, PPAR-α and PPAR-γ agonists have been shown to decrease iNOS, TNF-α, and IL-6 expression in different cell types including monocytes/macrophages (for review [66]). The present results, showing an attenuation of IFN-γ-induced up-regulation of TNF-α and iNOS by GW 501516 are in good agreement with these previous reports showing anti-inflammatory effects of PPAR agonists. These effects could be mediated through regulation of the NF-κB pathway, as it had been proposed previously [66].

GW 501516 also up-regulated the expression of PPAR-β and PPAR-γ. In good agreement with these findings, it was shown that GW 0742, another PPAR-β agonist, increased PPAR-β expression at the protein level [67], and that a PPAR-γ agonist up-regulated PPAR-γ mRNA expression [68,69]. The effects of GW 501516 on PPAR-γ expression could be due to a regulation of PPAR-β on PPAR-γ expression or to a residual affinity of this PPAR-β agonist for PPAR-γ.

The demyelinating treatment induced the reactivity of both microglial cells and astrocytes, whereas IFN-γ activated only microglial cells. On the other hand, the antibody-mediated demyelination, in contrast to IFN-γ stimulation, did not increase the expression of the inflammation-related genes TNF-α, IL-6, and iNOS. Polak and collaborators [43] reported that the PPAR-β agonist GW 0742 attenuated clinical symptoms of EAE, increased the expression of some myelin-specific genes, and decreased the LPS-induced up-regulation of iNOS in cultures of astrocytes and microglial cells. Since antibody-mediated demyelination did not upregulate the expression of pro-inflammatory cytokines and iNOS, it appears that in this particular demyelination paradigm no typical inflammatory responses occurred, despite the observed microglial and astroglial activation. The isolated microglial reaction could have been elicited by the presence of myelin debris. Interestingly, GW 501516 affected differently GFAP, IL-6, PPAR-β and PPAR-γ expression in controls compared to demyelinating cultures, while the effects of this PPAR-β agonist were similar to controls in the classical inflammatory response triggered by IFN-γ and LPS. Moreover, the treatment with GW 501516 did not protect against demyelination.

Our results suggest that the protective effects of PPAR-β agonists observed in other studies could be mainly due to their anti-inflammatory effects rather than to a direct protective effect on oligodendrocytes and myelin. In the EAE model of demyelination, Polak and collaborators showed that GW 0742 reduced the occurrence of small lesions in some brain regions, while it did not affect the number of cerebellar infiltrates. This suggests that the chronic treatment with GW 0742 inhibited a specific set of lymphocytes. The present *in vitro *model of demyelination is devoid of lymphocytes, thus inflammatory responses are limited to microglial cells and astrocytes. The present results are in agreement with the view that the protective effect of GW 501516 in the *in vivo *demyelinating model was due to its anti-inflammatory potency, and that it does not provide direct protective or trophic effects on oligodendrocytes or myelin.

## Competing interests

The authors declare that they have no competing interests.

## Authors' contributions

AD carried out most of the experimental and statistical work, and drafted the manuscript. FMT was instrumental in the conception and design of the study, and responsible for the immunocytochemical work. MGZ supervised the quantitative RT-PCR analyses. OB was responible for the analyses by *in situ *hybridization. PH was responsible for the cell culture work. All co-authors contributed to the preparation of the manuscript.

## References

[B1] Neumann H (2001). Control of glial immune function by neurons. Glia.

[B2] Glabinsk AR, Ransohoff RM (2001). Targeting the chemokine system for multiple sclerosis treatment. Curr Opin Investig Drugs.

[B3] Hemmer B, Cepok S, Nessler S, Sommer N (2002). Pathogenesis of multiple sclerosis: an update on immunology. Curr Opin Neurol.

[B4] Streit WJ, Walter SA, Pennell NA (1999). Reactive microgliosis. Prog Neurobiol.

[B5] Dong Y, Benveniste EN (2001). Immune function of astrocytes. Glia.

[B6] Cannella B, Raine CS (2004). Multiple sclerosis: cytokine receptors on oligodendrocytes predict innate regulation. Ann Neurol.

[B7] Juttler E, Tarabin V, Schwaninger M (2002). Interleukin-6 (IL-6): a possible neuromodulator induced by neuronal activity. Neuroscientist.

[B8] Panitch H (1992). Interferons in multiple sclerosis. A review of the evidence. Drugs.

[B9] Hogg N, Selvendran Y, Dougherty G, Allen C (1986). Macrophage antigens and the effect of a macrophage activating factor, interferon-gamma. Ciba Found Symp.

[B10] Nathan CF (1987). Secretory products of macrophages. J Clin Invest.

[B11] Woodroofe MN, Hayes GM, Cuzner ML (1989). Fc receptor density, MHC antigen expression and superoxide production are increased in interferon-gamma-treated microglia isolated from adult rat brain. Immunology.

[B12] Lin W, Kemper A, Dupree JL, Harding HP, Ron D, Popko B (2006). Interferon-gamma inhibits central nervous system remyelination through a process modulated by endoplasmic reticulum stress. Brain.

[B13] Diemel LT, Wolswijk G, Jackson SJ, Cuzner ML (2004). Remyelination of cytokine- or antibody-demyelinated CNS aggregate cultures is inhibited by macrophage supplementation. Glia.

[B14] Hagberg H, Mallard C (2005). Effect of inflammation on central nervous system development and vulnerability. Curr Opin Neurol.

[B15] Lund S, Christensen KV, Hedtjarn M, Mortensen AL, Hagberg H, Falsig J, Hasseldam H, Schrattenholz A, Porzgen P, Leist M (2006). The dynamics of the LPS triggered inflammatory response of murine microglia under different culture and in vivo conditions. J Neuroimmunol.

[B16] Lehnardt S, Lachance C, Patrizi S, Lefebvre S, Follett PL, Jensen FE, Rosenberg PA, Volpe JJ, Vartanian T (2002). The toll-like receptor TLR4 is necessary for lipopolysaccharide-induced oligodendrocyte injury in the CNS. J Neurosci.

[B17] Qin L, Wu X, Block ML, Liu Y, Breese GR, Hong JS, Knapp DJ, Crews FT (2007). Systemic LPS causes chronic neuroinflammation and progressive neurodegeneration. Glia.

[B18] Braissant O, Wahli W (1998). Differential expression of peroxisome proliferator-activated receptor-alpha, -beta, and -gamma during rat embryonic development. Endocrinology.

[B19] Braissant O, Foufelle F, Scotto C, Dauca M, Wahli W (1996). Differential expression of peroxisome proliferator-activated receptors (PPARs): tissue distribution of PPAR-alpha, -beta, and -gamma in the adult rat. Endocrinology.

[B20] Woods JW, Tanen M, Figueroa DJ, Biswas C, Zycband E, Moller DE, Austin CP, Berger JP (2003). Localization of PPARdelta in murine central nervous system: expression in oligodendrocytes and neurons. Brain Res.

[B21] Lim H, Gupta RA, Ma WG, Paria BC, Moller DE, Morrow JD, DuBois RN, Trzaskos JM, Dey SK (1999). Cyclo-oxygenase-2-derived prostacyclin mediates embryo implantation in the mouse via PPARdelta. Genes Dev.

[B22] Magge SS, Guardiola-Diaz HM (2002). Characterization of the mouse peroxisome proliferator-activated receptor delta gene. Biochem Biophys Res Commun.

[B23] Basu-Modak S, Braissant O, Escher P, Desvergne B, Honegger P, Wahli W (1999). Peroxisome proliferator-activated receptor beta regulates acyl-CoA synthetase 2 in reaggregated rat brain cell cultures. J Biol Chem.

[B24] Cimini A, Bernardo A, Cifone MG, Di Marzio L, Di Loreto S (2003). TNF alpha downregulates PPARdelta expression in oligodendrocyte progenitor cells: implications for demyelinating diseases. Glia.

[B25] Granneman J, Skoff R, Yang X (1998). Member of the peroxisome proliferator-activated receptor family of transcription factors is differentially expressed by oligodendrocytes. J Neurosci Res.

[B26] Saluja I, Granneman JG, Skoff RP (2001). PPAR delta agonists stimulate oligodendrocyte differentiation in tissue culture. Glia.

[B27] Roth AD, Leisewitz AV, Jung JE, Cassina P, Barbeito L, Inestrosa NC, Bronfman M (2003). PPAR gamma activators induce growth arrest and process extension in B12 oligodendrocyte-like cells and terminal differentiation of cultured oligodendrocytes. J Neurosci Res.

[B28] Peters JM, Lee SS, Li W, Ward JM, Gavrilova O, Everett C, Reitman ML, Hudson LD, Gonzalez FJ (2000). Growth, adipose, brain, and skin alterations resulting from targeted disruption of the mouse peroxisome proliferator-activated receptor beta(delta). Mol Cell Biol.

[B29] Berger J, Moller DE (2002). The mechanisms of action of PPARs. Annu Rev Med.

[B30] Daynes RA, Jones DC (2002). Emerging roles of PPARs in inflammation and immunity. Nat Rev Immunol.

[B31] Delerive P, Fruchart JC, Staels B (2001). Peroxisome proliferator-activated receptors in inflammation control. J Endocrinol.

[B32] Jiang C, Ting AT, Seed B (1998). PPAR-gamma agonists inhibit production of monocyte inflammatory cytokines. Nature.

[B33] Ricote M, Li AC, Willson TM, Kelly CJ, Glass CK (1998). The peroxisome proliferator-activated receptor-gamma is a negative regulator of macrophage activation. Nature.

[B34] Clark RB, Bishop-Bailey D, Estrada-Hernandez T, Hla T, Puddington L, Padula SJ (2000). The nuclear receptor PPAR gamma and immunoregulation: PPAR gamma mediates inhibition of helper T cell responses. J Immunol.

[B35] Henson P (2003). Suppression of macrophage inflammatory responses by PPARs. Proc Natl Acad Sci USA.

[B36] Storer PD, Xu J, Chavis J, Drew PD (2005). Peroxisome proliferator-activated receptor-gamma agonists inhibit the activation of microglia and astrocytes: implications for multiple sclerosis. J Neuroimmunol.

[B37] Storer PD, Xu J, Chavis JA, Drew PD (2005). Cyclopentenone prostaglandins PGA2 and 15-deoxy-delta12,14 PGJ2 suppress activation of murine microglia and astrocytes: implications for multiple sclerosis. J Neurosci Res.

[B38] Welch JS, Ricote M, Akiyama TE, Gonzalez FJ, Glass CK (2003). PPARgamma and PPARdelta negatively regulate specific subsets of lipopolysaccharide and IFN-gamma target genes in macrophages. Proc Natl Acad Sci USA.

[B39] Xu J, Storer PD, Chavis JA, Racke MK, Drew PD (2005). Agonists for the peroxisome proliferator-activated receptor-alpha and the retinoid X receptor inhibit inflammatory responses of microglia. J Neurosci Res.

[B40] Niino M, Iwabuchi K, Kikuchi S, Ato M, Morohashi T, Ogata A, Tashiro K, Onoe K (2001). Amelioration of experimental autoimmune encephalomyelitis in C57BL/6 mice by an agonist of peroxisome proliferator-activated receptor-gamma. J Neuroimmunol.

[B41] Feinstein DL, Galea E, Gavrilyuk V, Brosnan CF, Whitacre CC, Dumitrescu-Ozimek L, Landreth GE, Pershadsingh HA, Weinberg G, Heneka MT (2002). Peroxisome proliferator-activated receptor-gamma agonists prevent experimental autoimmune encephalomyelitis. Ann Neurol.

[B42] Duvanel CB, Honegger P, Pershadsingh H, Feinstein D, Matthieu JM (2003). Inhibition of glial cell proinflammatory activities by peroxisome proliferator-activated receptor gamma agonist confers partial protection during antimyelin oligodendrocyte glycoprotein demyelination in vitro. J Neurosci Res.

[B43] Polak PE, Kalinin S, Dello Russo C, Gavrilyuk V, Sharp A, Peters JM, Richardson J, Willson TM, Weinberg G, Feinstein DL (2005). Protective effects of a peroxisome proliferator-activated receptor-beta/delta agonist in experimental autoimmune encephalomyelitis. J Neuroimmunol.

[B44] Arsenijevic D, de Bilbao F, Plamondon J, Paradis E, Vallet P, Richard D, Langhans W, Giannakopoulos P (2006). Increased infarct size and lack of hyperphagic response after focal cerebral ischemia in peroxisome proliferator-activated receptor beta-deficient mice. J Cereb Blood Flow Metab.

[B45] Honegger P, Lenoir D, Favrod P (1979). Growth and differentiation of aggregating fetal brain cells in a serum-free defined medium. Nature.

[B46] Honegger P, Matthieu JM, Lassmann H (1989). Demyelination in brain cell aggregate cultures, induced by a monoclonal antibody against the myelin/oligodendrocyte glycoprotein (MOG). Schweiz Arch Neurol Psychiatr.

[B47] Kerlero de Rosbo N, Honegger P, Lassmann H, Matthieu JM (1990). Demyelination induced in aggregating brain cell cultures by a monoclonal antibody against myelin/oligodendrocyte glycoprotein. J Neurochem.

[B48] Honegger P, Monnet-Tschudi F (2001). Aggregating neural cell cultures.

[B49] Lowry OH, Rosebrough NJ, Farr AL, Randall RJ (1951). Protein measurement with the Folin phenol reagent. J Biol Chem.

[B50] Koh JY, Choi DW (1987). Quantitative determination of glutamate mediated cortical neuronal injury in cell culture by lactate dehydrogenase efflux assay. J Neurosci Methods.

[B51] Lengacher S, Magistretti PJ, Pellerin L (2004). Quantitative rt-PCR analysis of uncoupling protein isoforms in mouse brain cortex: methodological optimization and comparison of expression with brown adipose tissue and skeletal muscle. J Cereb Blood Flow Metab.

[B52] Livak KJ, Schmittgen TD (2001). Analysis of relative gene expression data using real-time quantitative PCR and the 2(-Delta Delta C(T)) Method. Methods.

[B53] Streit WJ, Kreutzberg GW (1988). Response of endogenous glial cells to motor neuron degeneration induced by toxic ricin. J Comp Neurol.

[B54] Ashwell K (1990). Microglia and cell death in the developing mouse cerebellum. Brain Res Dev Brain Res.

[B55] Escher P, Braissant O, Basu-Modak S, Michalik L, Wahli W, Desvergne B (2001). Rat PPARs: quantitative analysis in adult rat tissues and regulation in fasting and refeeding. Endocrinology.

[B56] Braissant O (2004). Measurement of nitric oxide-related enzymes in the brain by in situ hybridization. Methods Mol Biol.

[B57] Duvanel CB, Monnet-Tschudi F, Braissant O, Matthieu JM, Honegger P (2004). Tumor necrosis factor-alpha and alphaB-crystallin up-regulation during antibody-mediated demyelination in vitro: a putative protective mechanism in oligodendrocytes. J Neurosci Res.

[B58] Diemel LT, Jackson SJ, Cuzner ML (2003). Role for TGF-beta1, FGF-2 and PDGF-AA in a myelination of CNS aggregate cultures enriched with macrophages. J Neurosci Res.

[B59] Loughlin AJ, Honegger P, Woodroofe MN, Comte V, Matthieu JM, Cuzner ML (1994). Myelin basic protein content of aggregating rat brain cell cultures treated with cytokines and/or demyelinating antibody: effects of macrophage enrichment. J Neurosci Res.

[B60] Eskes C, Honegger P, Juillerat-Jeanneret L, Monnet-Tschudi F (2002). Microglial reaction induced by noncytotoxic methylmercury treatment leads to neuroprotection via interactions with astrocytes and IL-6 release. Glia.

[B61] Gruol DL, Nelson TE (1997). Physiological and pathological roles of interleukin-6 in the central nervous system. Mol Neurobiol.

[B62] Bissonnette CJ, Klegeris A, McGeer PL, McGeer EG (2004). Interleukin 1alpha and interleukin 6 protect human neuronal SH-SY5Y cells from oxidative damage. Neurosci Lett.

[B63] Taga T, Fukuda S (2005). Role of IL-6 in the neural stem cell differentiation. Clin Rev Allergy Immunol.

[B64] Penkowa M, Giralt M, Lago N, Camats J, Carrasco J, Hernandez J, Molinero A, Campbell IL, Hidalgo J (2003). Astrocyte-targeted expression of IL-6 protects the CNS against a focal brain injury. Exp Neurol.

[B65] Acarin L, Gonzalez B, Castellano B (2000). Neuronal, astroglial and microglial cytokine expression after an excitotoxic lesion in the immature rat brain. Eur J Neurosci.

[B66] Moraes LA, Piqueras L, Bishop-Bailey D (2005). Peroxisome proliferator-activated receptors and inflammation. Pharmacol Ther.

[B67] Yin Y, Russell RG, Dettin LE, Bai R, Wei ZL, Kozikowski AP, Kopelovich L, Glazer RI (2005). Peroxisome proliferator-activated receptor delta and gamma agonists differentially alter tumor differentiation and progression during mammary carcinogenesis. Cancer Res.

[B68] Panchapakesan U, Pollock CA, Chen XM (2004). The effect of high glucose and PPAR-gamma agonists on PPAR-gamma expression and function in HK-2 cells. Am J Physiol Renal Physiol.

[B69] Zurich MG, Lengacher S, Braissant O, Monnet-Tschudi F, Pellerin L, Honegger P (2005). Unusual astrocyte reactivity caused by the food mycotoxin ochratoxin A in aggregating rat brain cell cultures. Neuroscience.

